# Myocardial and vascular dysfunction in patients with rheumatoid arthritis: insights from cardiovascular magnetic resonance

**DOI:** 10.1186/1532-429X-15-S1-P221

**Published:** 2013-01-30

**Authors:** Ntobeko A Ntusi, Jane M Francis, Paul M Matthews, Paul B Wordsworth, Stefan Neubauer, Theodoros Karamitsos

**Affiliations:** 1 Cardiovascular Medicine, University of Oxford, Oxford, UK; 2 GSK Clinical Imaging Centre, Imperial College, London, UK; 3 Bortnar Institute and Nuffield Department of Orthopaedics, Rheumatology and Musculoskeletal Sciences, University of Oxford, Oxford, UK

## Background

Rheumatoid arthritis (RA) is a multi-system, autoimmune disorder and is one of the strongest known risk factors for cardiovascular disease (CVD) morbidity and mortality. Endothelial dysfunction, accelerated atherosclerosis, vascular inflammation and myocarditis are thought to contribute to this excess CVD. Cardiovascular magnetic resonance (CMR) has the capacity of simultaneously assessing non-invasively cardiac function, altered vascular distensibility, myocardial strain and fibrosis. The purpose of this study was to assess cardiac and vascular function and to determine their relation to the presence of cardiovascular risk factors (CVRFs) and RA disease duration.

## Methods

22 RA patients with no CVRFs (16 female, mean age 51 ± 13), 44 RA patients with CVRFs (31 female, mean age 53± 12), 35 normal controls (31 female, mean age 49 ± 10), and 18 controls with CVRFs (10 female, mean age 51± 11), underwent CMR at 1.5 Tesla. All patients with previously known CVD were excluded. CVRFs and duration of disease were recorded for each subject. Biventricular volumes and function, LGE, myocardial strain and vascular function were assessed by CMR. Aortic distensibility and pulse wave velocity were measured in the ascending aorta, proximal descending aorta and distal descending aorta.

## Results

There were no differences in left ventricular (LV) volumes and LV ejection fraction between the 4 groups (Table 1). RA patients with CVRFs showed the greatest reduction in mid short axis circumferential systolic strain, peak diastolic strain rate, and vascular indices. RA patients without CVRFs showed a similar degree of vascular dysfunction and deformational abnormality as controls with CVRFs (Table 2). Aortic distensibility (Rs= -0.25, p=0.048) and total pulse wave velocity (Rs= 0.41, p<0.001) correlated with RA disease duration.

**Table 1:** Demographic, clinical and CMR features in RA, RA with CVRFs, controls and controls with CVRFs.

	Normal controls N=35	Controls with CVRFs N=18	RA N=22	RA with CVRF N=44	P Value
Age (years)	51.2 ± 13.3	53.4 ± 11.8	49.2 ± 9.8	51.2 ± 10.7	0.19

Females (%)	21 (60.0)	10 (55.0)	16 (72.7)	31 (70.5)	0.06

BMI (kg/m2)	23.3 ± 2.7	27.3 ± 4.2	24.5 ± 2.8	28.4 ± 7.4	<0.001

LVEDV (ml) indexed to BSA	72.3 ± 12.2	73.0 ± 15.4	74.3 ± 19.4	10.1 ± 14.2	0.72

LVESV (ml) indexed to BSA	20.7 ± 13.2	18.6 ± 5.3	21.9 ± 9.3	19.5 ± 8.3	0.65

LVEF	73.8 ± 4.5	74.5 ± 5.2	71.2 ± 5.7	72.9 ± 7.1	0.17

LA size	2.7 ± 0.5	3.0 ± 0.6	3.2 ± 0.5	3.2 ± 0.6	<0.001

BME, Body mass index; LVEDV, Left ventricular end diastolic volume; BSA, Body surface area; LVESV, Left ventricular end systolic volume; LVEF, Left ventricular ejection fraction; LA, Left atrium.

**Table 2:** Systolic circumferential strain, aortic distensibility and pulse wave velocity in RA, RA with CVRFs, controls and controls with CVRFs.

	Normal controls N=35	Controls with CVRFs N=18	RA N=22	RA with CVRFs N=44	P Value
Mid SA systolic circumferential strain	-19.2 ± 1.1	-17.6 ± 1.2	-17.26 ± 1.4	-16.9 ± 1.2	<0.001

Peak diastolic strain rate	143.9 ± 19.7	113.6 ± 27.9	101.9 ± 23.5	103.1 ± 20.3	<0.001

Aortic distensibility (10-3mmHg-1) Ascending aorta Proximal descending aorta Distal descending aorta	3.2 ± 1.8 3.7 ± 1.3 5.7 ± 2.0	2.1 ± 1.5 3.1 ± 1.4 4.5 ± 1.5	2.2 ± 1.5 2.7 ± 1.2 4.0 ± 1.5	2.0 ± 1.3 2.1 ± 1.5 3.6 ± 1.7	0.002 <0.001 <0.001

Pulse wave velocity (m/s) Aortic arch PWV Descending aorta PWV Total PWV	4.2 ± 2.6 3.8 ± 1.5 4.4 ± 1.6	6.2 ± 1.9 6.2 ± 1.8 6.7 ± 1.5	6.8 ± 3.1 6.9 ± 2.9 7.7 ± 2.9	7.2 ± 2.2 7.7 ± 2.3 8.3 ± 2.8	<0.001 <0.001 <0.001

SA, short axis; PWV, pulse wave velocity.

## Conclusions

CMR demonstrates impaired myocardial deformational characteristics and impaired vascular function in RA and in patients with CVRFs. The cardiac abnormalities due to RA appear to be independent and incremental to those due to traditional CVRFs.

## Funding

NN is funded by the Discovery Foundation and the Nuffield Trust. SN acknowledges support from the British Heart Foundation Centre of Research Excellence, Oxford.The research was funded through an investigator-led grant from GSK.

